# Predictors of 1-year mortality in a clinical cohort of hip fracture patients

**DOI:** 10.1007/s00068-025-02812-y

**Published:** 2025-03-20

**Authors:** Mads Sundet, Mette Martinsen, Maren Paus, Haldor Valland, Henriette Haugeli Halvorsen, Joseph Sexton, Ulf Sundin, Siri Lillegraven

**Affiliations:** 1 Department of Orthopedic Surgery, Diakonhjemmet Hospital, Vinderen, Box 23, 0319 Oslo, Norway; 2 Center for Treatment of Rheumatic and Musculoskeletal Diseases (REMEDY), Diakonhjemmet Hospital, Oslo, Norway; 3 Faculty of Medicine, University of Oslo, Oslo, Norway

**Keywords:** Hip fracture, Predictors, Mortality, Obesity paradox

## Abstract

**Purpose:**

Knowledge about factors associated with mortality after hip fracture is important both for analytical and clinical purposes. This study aimed to assess patient risk factors and commonly used composite scores for prediction of 1-year mortality in a large clinical cohort.

**Methods:**

Hip fracture patient data were prospectively recorded in a local hospital database. Consecutive fractures from 2006 to 2020 were included, 6040 fractures in 5496 patients. Associations between 1-year mortality and different exposures were estimated using univariate and two multivariate logistic regression models. ROC analysis was used to compare the ability of the Nottingham Hip Fracture Score (NHFS), Age-adjusted Charlson Comorbidity Index (ACCI) the American Society of Anesthesiologists score (ASA) and the Orthopedic Frailty Score (OFS) to predict 1-year mortality.

**Results:**

Females sustained 73.9% of the fractures. Total 1-year mortality was 24.8%. Patients with overweight and class 1 obesity had lower 1-year mortality rates than normal weight patients [overweight: adjusted OR 0.58 (0.45–0.77), class 1 obesity: adjusted OR 0.40 (0.21–0.75)]. Mortality was elevated in males (adjusted OR 2.04, 95% CI 1.76–2.36), and nursing home residents (adjusted OR 2.99, 95% CI 2.60–3.44). We found no significant association between waiting time before surgery and mortality. Models including ACCI (AUC 0.74), NHFS (AUC 0.75) and OFS (AUC 0.73) had a similar ability to predict 1-year mortality, while a model including ASA (AUC 0.71) had a significantly lower prediction ability than ACCI and NHFS.

**Conclusions:**

Sex, age, cognitive impairment, and residential status predicted 1-year mortality. The study found an apparent “obesity paradox”, where overweight patients had a lower mortality rate than normal weight patients, but unmeasured confounding may have biased this analysis. ACCI and NHFS predicted mortality better than the combination of age, sex, and ASA.

## Introduction

Hip fractures are a major cause of morbidity and mortality in the elderly population. Globally, the 1-year mortality rate is estimated at 22%, but there are large regional differences [1, 2]. The incidence rates of hip fractures in the Scandinavian countries are among the highest in the world [3].

Knowledge about factors associated with mortality in hip fracture patients is important for several reasons. Modifiable risk factors (e.g. waiting time before surgery) can be addressed [4, 5], and information about non-modifiable risk factors (e.g. sex) can point out avenues for further research and interventions [6]. As most studies on hip fracture treatment are observational and not randomized, appropriate adjustment for confounding factors (e.g. comorbidities) in the data analysis is essential to minimize bias, which may cause misleading results and conclusions. Several measures and instruments are currently used to measure comorbidity, and it is important to investigate the ability of these instruments to predict mortality. A review of the literature found wide agreement about several risk factors associated with increased mortality following hip fracture: ASA score, male sex, age, cognitive impairment and nursing home residency, and diverging findings about the effect of preoperative waiting time [7].

The aim of the present study was to estimate the association between several tentative risk factors and 1-year mortality in a large clinical cohort. We also wanted to compare the performance of the ASA score, the age-adjusted Charlson Comorbidity Index (ACCI) [8] the Nottingham Hip Fracture Score (NHFS) [9, 10], and the Orthopedic Frailty Score (OFS) in the prediction of 1-year mortality [11, 12].

## Materials and methods

### The Diakonhjemmet Hip Fracture Registry

Since 2006, data on hip fracture cases treated at Diakonhjemmet Hospital have been collected in the Diakonhjemmet Hip Fracture Registry (DHFR). Until 2015, only patients aged ≥ 65 years were recorded, since then all patients have been registered regardless of age. The database contains data on comorbidities, medication, treatment, complications, demographic information, laboratory tests, fracture classification, operation type, ASA-class, time of surgery, and length of hospital stay. Information about deaths is retrieved from the Norwegian National Population Register and entered into the DHFR database yearly. This study includes all registered cases from 2006 to 2020, 6040 hip fractures in 5649 patients. Patients admitted in 2021 and 2022 were excluded to take into account possible delays in the transfer of information between registries.

The register has recorded all the components of the ACCI, which is based on 17 weighted disease categories. Registrations of ACCI prior to 2011 has been set as missing as the recording was only sporadic in this time period. The NHFS was calculated when information required for this score was available, i.e. admission blood hemoglobin concentration (g/L), age, sex, cognitive status, number of comorbidities, nursing home residency, and malignancies. The NHFS utilizes the Abbreviated Mental Test Score for a dichotomous recording of cognitive impairment. This score was not available in our dataset, and this was substituted with the clinical diagnosis of cognitive impairment that was registered in our database. This diagnose was either established prior to admission to hospital, or made during the hospital stay. The Orthopedic Frailty Score (OFS) was calculated using information about age, heart failure, malignancies, nursing home residence and functional status [12]. The diagnosis of cognitive impairment was used as a surrogate for functional status. Other measures of frailty and sarcopenia were not registered.

### Outcome and exposure variables

The outcome in this study was death within 1 year after admission for a hip fracture (1-year mortality). The association between 1-year mortality and the following exposures were analyzed: age, sex, BMI, cognitive impairment, heart failure, nursing-home residency, time period of surgery (3-year periods), fracture type, waiting time before surgery, type of surgery, NHFS, ACCI, OFS and ASA score.

### Statistical analysis

Each hip fracture and subsequent mortality outcome was analyzed as a separate case without adjustment for dependency of data. Demographic and background factors were described by means (95% CI) or frequencies (percent) as appropriate, grouped by the outcome (death within 1 year after admission). Associations between the outcome and exposure factors were first estimated by univariable logistic regression analyses. The literature was reviewed to determine which variables to adjust for [5, 7, 13, 14] and based on this, two multivariable models were constructed; one adjusting for age groups and sex (model 1), and one with age group, sex, ASA-score, and residential status (nursing home/community) as covariates (model 2). We chose to use ASA score as the adjustment covariate for comorbidity, as we had almost complete data for this variable. Because the correlation of ASA and age groups on mortality was non-linear, these were analyzed as categorical variables. The most common category was set as the reference for categorical variables. For the categorical variables with an ordinal structure, the lowest value was set as the reference value, except for BMI where “normal weight” (BMI 18.5–25) [15] was set as the reference. Analyses were not corrected for multiple testing. In one of the adjustment models (model 2) there were variables with missing information (2% missing in residential status, 1% missing in ASA score). A sensitivity analysis was performed excluding the patients with these missing covariates from model 1, and results were compared to identify potential relevant changes in the effect estimates. In the analysis of BMI and mortality, an extra model was fitted (model 3) where ACCI also was included in the model to ensure better adjustment for comorbidity. The collinearity between ASA and ACCI in this model was acceptable. In the patients with recorded BMI, 23% had missing registrations for ACCI, and multiple imputations were performed for these missing values, detailed in the next section.

To assess the performance of different measures of comorbidity for adjustment of predictive models, we performed receiver operating characteristics (ROC) analysis for the different measures of comorbidity (NHFS, ACCI, OFS and ASA) based on a logistic regression with 1-year mortality as the outcome. As the NHFS has both age and sex integrated, sex was also included as a covariate in the ACCI and OFS-models and both age and sex were included in the ASA model. The statistical significance of differences between AUCs of different models were tested with the *rocgold* and *roccomp* commands in Stata, which are based on non-parametric methodology described by Delong, Delong and Clarke-Pearson [16].

All analyses were done using Stata version 17.0 (StataCorp, College Station, Tx, USA).

### Missing information

Since the DHFR was launched in 2006, data collection has been revised on several occasions. Until 2010, cognitive impairment was not recorded. Hemoglobin concentration at admission was not recorded systematically in the database prior to 2017, and consequently, NHFS is only calculated in cases after this time point. Components of ACCI have only been reliably recorded since 2011, and recordings before that were set as missing. When information was missing for conditions needed for the calculation of the ACCI and OFS scores, the condition was assumed as absent. The uni- and multivariable analyses of risk factors only included observations where the risk factor of interest was present. The adjustment covariates in the multivariable models had near-complete data (2% missing for residential status, 1% missing for ASA score), except in model 3 where ACCI was missing in 23% of the patients. Multiple imputation was performed for the variables involved in model 3, using the chained equations approach to generate 5 imputed datasets. ROC-analyses of different prediction models were performed on a sub-set of observations (n = 1825) admitted after 2017, where all compared scores (NHFS, ACCI, OFS and ASA) were available.

## Results

We recorded 6040 hip fractures in 5496 patients during the registration period (571 patients had two hip fractures recorded). In 1496 (24.8%) of the cases, the patient died within a year after admission. The mean age was 84.0 years (SD 8.9), and 73.9% of the patients were female (Table 1). Males were on average 2.8 years younger than females (95% CI 2.3–3.3).

**Table 1:** Overview of the population and the extent of missing data

	Total	Died within 1 year	
		No	Yes
Total	6040	4544 (75.2)	1496 (24.8)
Age			
Mean (95% CI)	83.9 (83.7–84.2)	82.8 (82.6–83.1)	87.4 (87.0–87.8)
Sex n (%)			
Female	4464 (73.9)	3487 (76.7)	977 (65.4)
Male	1576 (26.1)	1058 (23.3)	518 (34.6)
Fracture type, n (%)			
Neck of femur	3422 (56.7)	2608 (57.4)	814 (54.4)
Trochanteric	2217 (36.7)	1640 (36.1)	577 (38.6)
Subtrochanteric	384 (6.4)	286 (6.3)	98 (6.6)
Missing	17 (0.3)	11 (0.2)	6 (0.5)
Cognitive impairment, n (%)			
No	3410 (56.5)	2739 (60.3)	671 (44.9)
Yes	1208 (20.0)	740 (16.29)	467 (31.22)
Missing	1423 (23.6)	1061 (23.4)	355 (23.9)
Body mass index (BMI)			
Mean (95% CI)	22.7 (22.5–22.8)	22.9 (22.7–23.1)	21.6 (21.3–22.0)
Less than 18.5	599 (9.3)	388 (8.5)	171 (11.4)
18.5–25	2232 (37.0)	1803 (39.7)	429 (28.7)
25–30	732 (12.1)	643 (14.1)	89 (6.0)
30–35	140 (2.3)	127 (2.8)	13 (0.9)
Over 35	40 (0.7)	32 (0.7)	8 (0.5)
Missing	2338 (38.7)	1551 (52.5)	786 (52.5)

**Fig. 1:**
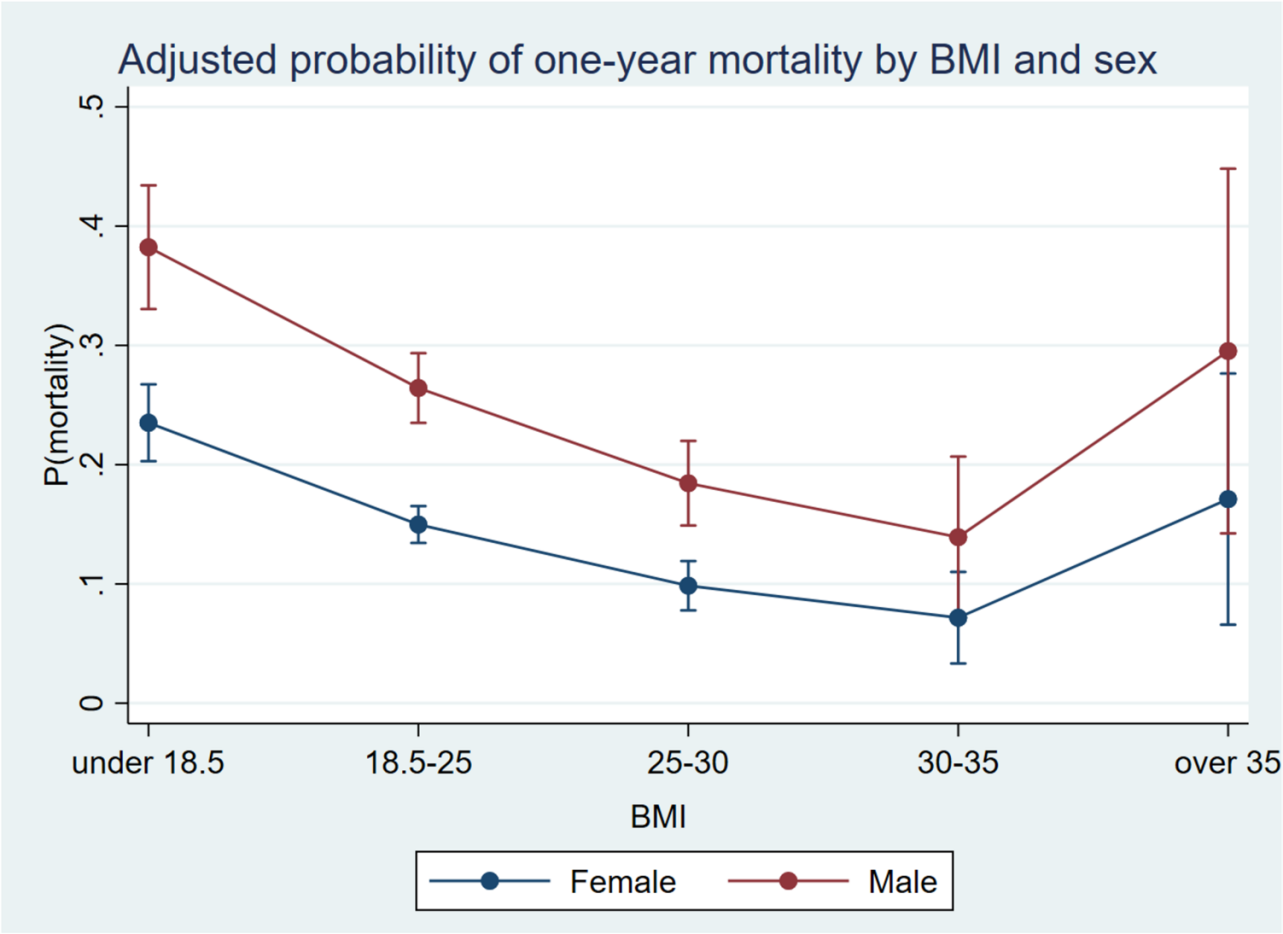
Representation of the regression results for the probability of mortality, by BMI-group and sex

Table 1 shows baseline data in the population, including the extent of missing data. Male sex and increasing age were associated with increasing mortality, and cases operated during the years 2012–2014 also had increased mortality compared to cases operated during other time periods, but this was not significant in adjustment model 2 (Table 2). In the unadjusted analysis, trochanteric fractures had a higher mortality, but when adjusted for relevant covariates the effect decreased and was non-significant. Males had slightly higher average BMI than women (0.98 units, 95% CI 0.65–1.31). The data show non-linear association between BMI and mortality, where a higher BMI is associated with a significantly decreasing risk of mortality up to a BMI of 35 (Table 3). The lowest mortality was observed for both men and women in the BMI 30–35 group (Fig. 1). No significant association was found between waiting time and mortality in adjusted or unadjusted models (Table 4). Operation with total hip arthroplasty was associated with a significantly lower mortality rate, also after adjusting for age, sex, ASA class, and residential status. Increasing NHFS, ACCI, OFS and ASA scores were all strongly associated with 1-year mortality (Table 5). A sensitivity analysis showed no relevant differences in the effect estimates when excluding patients with missing information in the model 2 analysis from the model 1 analysis.

**Table 2:** Logistic regression analysis of demographic exposures and fracture types

	N (% of total)	Died within 1 year (% within subcategory)	Crude OR (95% CI)*	Adjusted for sex and age groups (model 1)*	Adjusted for sex, age group, ASA class and residential status (model 2)*
Sex					
Female	4464 (73.9)	977 (21.9)	1	1	1
Male	1576 (26.1)	518 (32.9)	**1.75 (1.54–1.98)**	**2.15 (1.88–2.46)**	**2.04 (1.76–2.36)**
Age groups					
< 60	80 (1.3)	5 (6.3)	1	1	1
61–70	458 (7.6)	46 (10.0)	1.67 (0.64–4.35)	1.83 (0.70–4.78)	1.23 (0.46–3.26)
71–80	1253 (20.8)	193 (15.4)	**2.73 (1.09–6.84)**	**3.04 (1.21–7.66)**	1.50 (0.58–3.84)
81–90	2803 (46.4)	683 (24.4)	**4.83 (1.95–12.00)**	**5.86 (2.35–14.62)**	2.39 (0.94–6.08)
91 +	1446 (23.9)	568 (39.3)	**9.70 (3.90–24.14)**	**12.55 (5.01–31.44)**	**4.27 (1.67–10.92)**
Residential status					
Community dwelling	4476 (75.8)	746 (16.7)	1	1	1
Nursing home	1428 (24.2)	614 (43.0)	**3.77 (3.31–4.30)**	**3.29 (2.87–3.78)**	**2.99 (2.60–3.44)**
Operation period					
2006–2008	775 (12.8)	190 (24.5)	1	1	1
2009–2011	1123 (18.6)	298 (26.5)	1.11 (0.90–1.37)	1.10 (0.88–1.37)	1.09 (0.86–1.38)
2012–2014	1352 (22.4)	394 (29.1)	**1.27 (1.04–1.55)**	**1.25 (1.01–1.53)**	1.23 (0.98–1.55)
2015–2017	1468 (24.3)	314 (21.4)	0.84 (0.68–1.03)	0.84 (0.68–1.04)	0.75 (0.60–0.95)
2018–2020	1320 (21.9)	299 (22.7)	0.90 (0.73–1.11)	0.93 (0.75–1.16)	0.86 (0.68–1.09)
Fracture type					
Neck of femur	3423 (56.8)	814 (23.8)	1	1	1
Trochanteric	2217 (36.8)	577 (26.0)	**1.14 (1.01–1.26)**	1.07 (0.94–1.21)	1.04 (0.91–1.19)
Subtrochanteric	384 (6.4)	98 (25.5)	1.17 (0.67–2.05)	1.14 (0.64–2.03)	1.03 (0.55–1.92)

*Statistically significant results are printed with bold font

**Table 3:** Logistic regression analysis of BMI and comorbidities

	N (% of total)	Died within 1 year (% within subcategory)	Crude OR	Adjusted for sex and age groups (model 1)*	Adjusted for sex, age group, ASA class and residential status (model 2)*	Adjusted for sex, age group, ASA class, ACCI and residential status (model 3)*
BMI						
Less than 18.5	599 (15.1)	171 (30.6)	**1.85 (1.50–2.28)**	**2.01 (1.61–2.51)**	**1.89 (1.50–2.40)**	**1.93 (1.53–2.44)**
18.5–25	2232 (60.3)	429 (19.2)	**1**	**1**	**1**	**1**
25–30	732 (19.8)	89 (12.2)	**0.58 (0.46–0.74)**	**0.61 (0.48–0.79)**	**0.58 (0.45–0.77)**	**0.56 (0.43–0.73)**
30–35	140 (3.8)	13 (9.3)	**0.43 (0.24–0.77)**	**0.49 (0.27–0.90)**	**0.40 (0.21–0.75)**	**0.34 (0.18–0.65)**
35–	40 (1.1)	8 (20.0)	1.05 (0.48–2.30)	1.51 (0.66–3.42)	1.19 (0.51–2.82)	1.05 (0.44–2.51)
Heart failure						
No	3339 (86.2)	742 (22.2)	1	1	1	
Yes	534 (13.8)	232 (43.5)	**2.69 (2.22–3.26)**	**2.22 (1.82–2.70)**	**1.79 (1.43–2.24)**	
Cognitive impairment						
No	3416 (73.9)	673 (19.7)	1	1	1	
Yes	1208 (26.1)	467 (38.7)	**2.57 (2.23–2.97)**	**2.36 (2.03–2.74)**	**1.48 (1.21–1.80)**	

*Statistically significant results are printed with bold font

**Table 4:** Logistic regression analysis of waiting time and operation time

	N (% of total)	Died within 1 year (% within subcategory)	Crude OR*	Adjusted for sex and age groups*	Adjusted for sex, age group, ASA class and residential status (model 2)*
Waiting time					
Less than 24 h	4485 (74.6)	1090 (24.3)	1	1	1
24–48 h	1368 (22.8)	339 (24.8)	1.03 (0.89–1.18)	1.01 (0.87–1.16)	0.95 (0.81–1.11)
48–72 h	140 (2.3)	44 (31.4)	1.42 (0.99–2.05)	1.38 (0.94–2.01)	1.19 (0.79–1.79)
72 + h	20 (0.3)	7 (35.0)	1.68 (0.67–4.21)	1.92 (0.74–4.98)	1.15 (0.39–3.38)
Operation type					
Hemiarthroplasty	2425 (40.5)	621 (25.6)	1	1	1
Total arthroplasty	108 (1.8)	2 (1.9)	**0.06 (0.01–0.22)**	**0.11 (0.03–0.44)**	**0.18 (0.04–0.74)**
Fixation with screws	774 (12.9)	167 (21.6)	**0.80 (0.66–0.98)**	0.89 (0.72–1.09)	0.95 (0.76–1.18)
Sliding hip screw	1925 (32.1)	502 (26.1)	1.02 (0.89–1.17)	1.00 (0.87–1.15)	1.01 (0.87–1.19)
Intramedullar Nail	762 (12.7)	182 (23.9)	0.91 (0.75–1.10)	0.90 (0.74–1.09)	0.86 (0.70–1.07)

*Statistically significant results are printed with bold font

**Table 5:** 1-year mortality risk for four different measures of comorbidity

	N (% of total)	Died within 1 year (% within subcategory)	Crude OR for death*
Nottingham Hip Fracture Score			
0–2	85 (4.6)	6 (7.1)	1
3–4	839 (45.6)	70 (8.3)	1.2 (0.5–2.8)
5–6	684 (37.3)	199 (29.1)	**5.4 (2.3–12.6)**
7 +	226 (12.3)	110 (48.7)	**12.5 (5.2–29.8)**
ASA			
1	101 (1.7)	4 (4.0)	1
2	2410 (40.3)	342 (14.2)	**4.0 (1.5–11.0)**
3	3304 (55.2)	1026 (31.1)	**10.9 (4.0–29.8)**
4 +	172 (2.9)	104 (60.5)	**37.1 (13.0–105.5)**
Age-adjusted Charlson Comorbidity Index			
0–2	204 (4.4)	6 (2.9)	1
3–4	1162 (25.3)	94 (7.9)	**2.8 (1.2–6.6)**
5–6	2065 (44.9)	519 (25.1)	**11.1 (4.9–25.1)**
7–8	863 (18.8)	335 (38.8)	**20.9 (9.2–47.7)**
8–9	145 (3.2)	79 (54.5)	**39.5 (16.5–94.8)**
10 +	161 (3.5)	95 (59.0)	**47.5 (19.9–113.5)**
Orthopedic Frailty Score			
0	1792 (30.3)	130 (7.2)	**1**
1	2198 (37.2)	464 (21.1)	**3.4 (2.8–4.2)**
2	1150 (19.5)	422 (36.7)	**7.4 (6.0–9.2)**
3	634 (10.7)	265 (41.8)	**9.2 (7.2–11.7)**
4 +	131 (2.2)	79 (60.3)	**19.4 (13.1–28.8)**

*Statistically significant results are printed with bold font

**Fig. 2:**
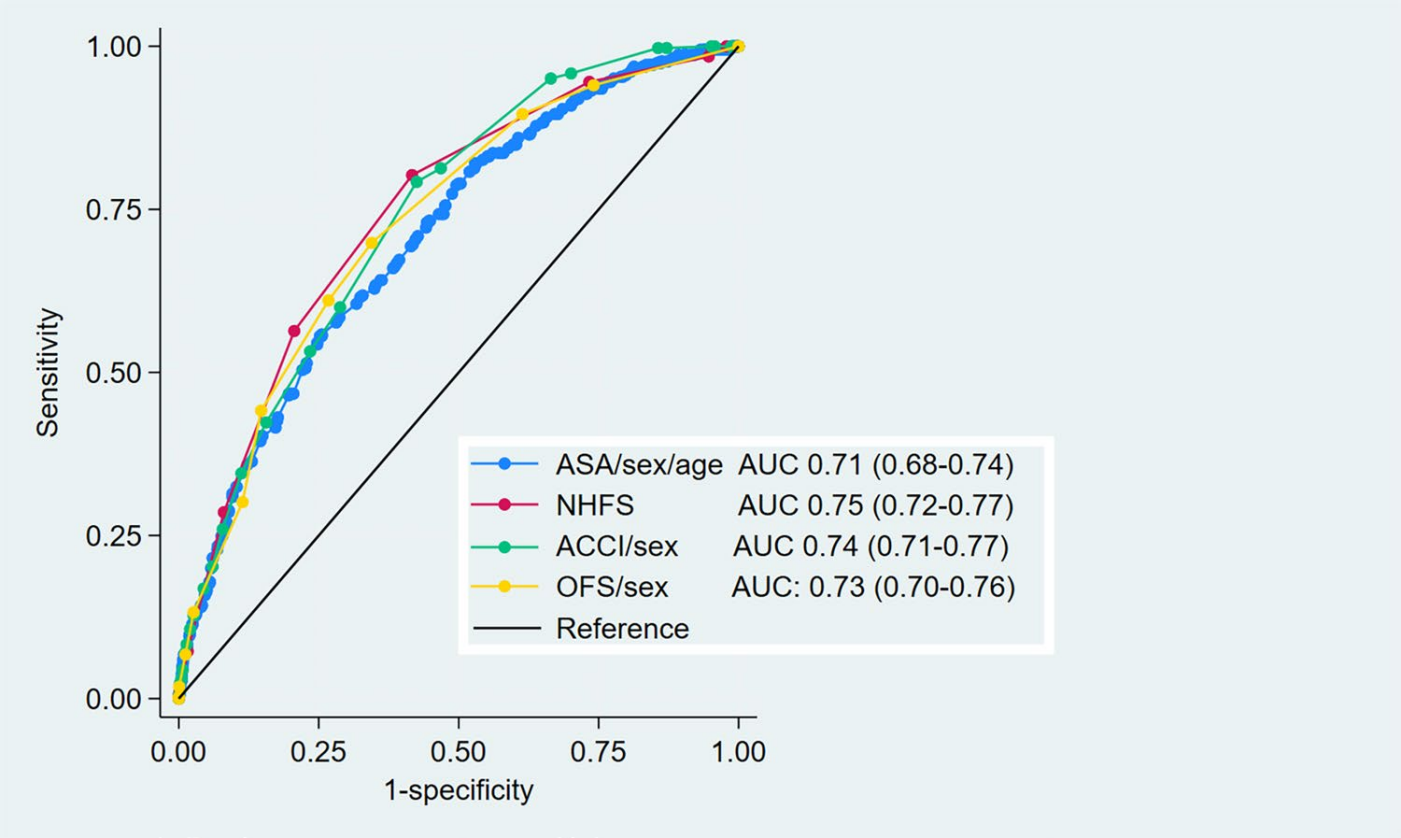
ROC-curves for the predictive abilities of 1-year mortality for the different comorbidity measures with appropriate covariates

**Fig. 3:**
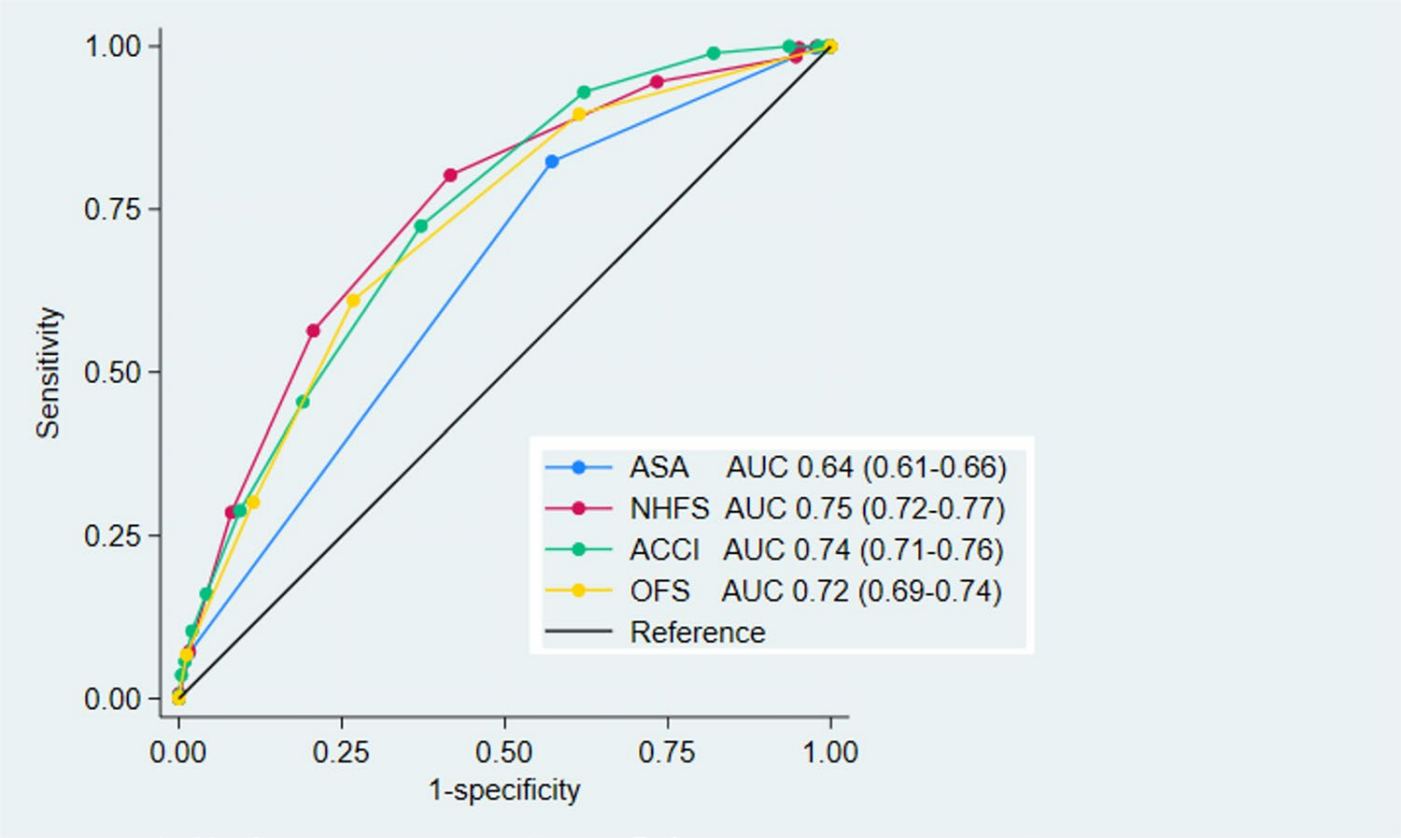
ROC-curves for the predictive abilities of 1-year mortality for the different comorbidity measures without covariates

When assessing the relationship between different measures of comorbidity and mortality, the three best models (NHFS, ACCI/sex and OFS/sex) had a very similar predictive ability for 1-year mortality (Fig. 2), and these models were not significantly different from each other using chi square tests between the individual models. The model including ASA, age and sex had a lower AUC at 0.71, which was significantly lower than the ACCI/sex model (p = 0.02) and the NHFS model (p = 0.01), but it was not significantly lower than the OFS/sex model (p = 0.12). Figure 3 visualizes the predictive abilities of the same comorbidity scores without any additions of age or sex as covariates. In this analysis, ASA clearly had a lower predictive ability than OFS (p < 0.001), NHFS predicted significantly better than OFS (p < 0.01) but similar to ACCI (p = 0.43). ACCI did not predict mortality better than OFS (p = 0.21). Table 6 summarizes the AUC of the different comorbidity measures, and the results of a non-parametric statistical analysis with NHFS set as the reference.

**Table 6:** Overview of the predictive abilities of comorbidity scores, with and without sex and age as additional covariates

	Scores without additional covariates			Models including additional covariates (age/sex as appropriate)		
	AUC	95% CI	p-Value	AUC	95% CI	p-Value
NHFS	0.745	0.72–0.77	Reference	0.745	0.72–0.77	Reference
ACCI	0.735	0.71–0.76	0.43	0.740	0.71–0.77	0.66
OFS	0.716	0.69–0.74	< 0.01	0.730	0.70–0.76	0.07
ASA	0.641	0.61–0.66	< 0.001	0.710	0.68–0.74	< 0.01

p-Values are from a non-parametric analysis [16] with NHFS set as reference

## Discussion

This study demonstrated several risk factors for 1-year mortality, including male sex, underweight and normal weight, nursing home residency, and high age. Comorbidity scores were found to predict mortality, with results indicating that NHFS, ACCI and OFS had better predictive abilities than a model with ASA as a comorbidity measure.

These findings are in accordance with previously published results [7]. A study of more than 37,000 hip fracture cases from the Norwegian Hip Fracture registry had similar findings, but they also found increased mortality in patients from a low income households, and patients with a lower educational level [5], variables that are not recorded in our database.

We found that the mortality in cases with a BMI classified as overweight or class 1 obesity [15], was half that of the cases classified as normal weight. This is often called an “obesity paradox”: that despite obesity being generally understood as a risk factor for death and complications, patients with obesity are frequently reported to have lower mortality than non-obese patients, both from medical and surgical conditions [17]. The obesity paradox has also previously been reported for hip fractures [13, 18]. The detrimental effect of being underweight in this study is not surprising, and can be related to factors such as comorbidities, malignancies, smoking and malnutrition. There were few patients with a class 2 or 3 obesity (BMI over 35), but these had no significant differences in mortality from the normal weight group.

The obesity paradox is poorly understood and controversial, and some epidemiologists hypothesize that the paradox is a mostly a product of biases, such as smoking [19]. In our study, smoking history was not available and could thus not be assessed in the analyses. The effect of smoking is expected to be somewhat limited as only 12% of the general population between 65 and 74 were reported to be current smokers in 2021 [20]. Another bias is that “normal” weight is in fact not the most common weight category in Norway; the majority of the Norwegian healthy population > 60 is overweight or obese [21]. This means that there may be an increased prevalence of malnutrition, malignancies and chronic diseases in the *normal weight* population compared to the overweight population. In a generally overweight population, a normal weight BMI in a patient may represent malnutrition and previous pathological weight loss from an obese state [22], and this is a potential explanation of the increased mortality in the normal weight group. Also, both frailty and sarcopenia are important risk factors for death after a hip fracture [23, 24], and measures reflecting these factors are not recorded in this data material. Frail and sarcopenic patients are more likely to have a normal or low BMI [25, 26], and this is probably one of the explanations of the “paradox”. Another unmeasured factor is the use of beta-blocker therapy, which has been shown to decrease mortality in hip fracture patients [27, 28]. As obesity is a risk factor for hypertension, is not unlikely that more patients in the high BMI groups are using beta-blockers, further explaining the decreased mortality.

There was an increased mortality during the years 2012–2014, although not statistically significant in adjustment model 2. We have no clear explanation for this finding, but it is interesting to note that from 2012 the Norwegian government introduced a reform (“The Coordination reform”), where the municipalities were obliged to take responsibility for hospitalized patients at a much earlier stage, leading to a decreased length of stay at the hospital and an earlier transfer of hip fracture patients to nursing homes and to home care services. Concerns were raised whether the resources and expertise in the municipal services were adequate for the treatment of these patients [29], but we have found no other publication documenting an increased mortality during this period. We plan to investigate this further.

Males had a considerably higher risk of death within 1 year after a hip fracture. The reason for this is unclear, and probably multifactorial. In our analysis factors such as age and ASA-class were adjusted for, but it is reasonable to suspect that there is unmeasured confounding related to comorbidity. Due to the positive effect of male sex hormones on bone structure, osteoporosis is more commonly affecting females, and it may be suspected that males who suffer hip fractures have other comorbidities affecting bone quality, which may not be reflected in their ASA-class. Other studies have also shown that this sex difference remains after controlling for medications and comorbidities [30]. Men generally live shorter than women due to a whole range of biological mechanisms [31], and most likely this also contributes to the sex differences in 1-year mortality after hip fracture.

Age is an important risk factor for death after hip fractures. In this study, the risk increase is large in the unadjusted analyses, but smaller in the analyses adjusted for sex, ASA and residence. When taking these factors into account, the increased mortality relative to patients under 60 is not significant before the patients are more than 90 years old, despite that the numbers are large. The interpretation of this might be that comorbidity (high ASA, nursing home residence) is what matters most (in addition to sex), not necessarily the chronological age. In other words: a previously healthy, community-dwelling (female) person have an excellent prognosis after a hip fracture even if the chronological age is high.

We did not find any increased risk of mortality in cases waiting between 24 and 48 h for surgery after admission. Similar findings are reported from the Norwegian Arthroplasty Register [32]. We also did not find any significant differences in mortality in cases waiting even longer than that, but in this group the numbers are too small to draw conclusions. Patients who wait more than 48 h usually are more comorbid, as seen in the differences between the unadjusted and adjusted analysis. Many of them wait because of preoperative optimalisation, and many of these patients also use direct-acting oral anticoagulants (DOACs). In our hospital, DOAC users wait until 48 h after DOAC-intake to allow for spinal anesthesia if general anesthesia is contraindicated because of comorbidities. Unfortunately, data about DOAC use in our patients was not available for most of this time period, and this was not included in the present analysis, but the impact of DOACs on waiting time before surgery in our hospital has been addressed in another publication [33].

Several measures of comorbidity are available for hip fracture patients, and they are important as adjustment factors to allow for comparisons between different risk factors, treatments or other exposures. In this study, NHFS, ACCI and OFS showed a similar ability to predict mortality, while ASA performed slightly worse than NHFS and ACCI. The ACCI contains information about previous medical history and demands a thorough file review or retrieval of information from patient registries. The NHFS and the OFS are simpler scores, specifically developed to predict mortality in hip fracture patients, the former utilizing information about age, sex, cognitive impairment, whether the patient have more than one comorbidity, residence, hemoglobin concentration, and malignancy, while the latter uses information about age, malignancies, institutional residency, congestive heart failure and non-independent functional status. These measures are substantially easier to obtain than the ACCI, both in prospective and in retrospective studies. The simple ASA classification that is designated to all surgical patients during pre-operative assessment is the normally the easiest score to obtain. Although the NHFS and ACCI/sex were significantly superior to the prediction model including age, sex and ASA, the differences were quite small, suggesting that ASA as a measure of comorbidity in hip fracture patients is a reasonable alternative that requires fewer resources.

### Strengths and limitations

There was a large proportion of missing values for BMI and cognitive impairment, and this may have affected the results. We found that patients with cognitive impairment had a higher proportion of missing values for BMI, most likely because they are not able to report their own weight and height, and obtaining objective measurements is often not prioritized during the hospital stay. It is not clear how these issues of missing values affect the results, but a selection bias cannot be ruled out as a partial explanation for our results. Patients with two consecutive fractures were analyzed as two separate cases, which may introduce a bias as these recordings are not independent. The database also has no information about smoking history, which may be an unmeasured confounder. The calculation of OFS and ACCI had methodological weaknesses as conditions were considered absent when registrations were missing, and for OFS cognitive impairment was used as a surrogate for non-independent functional status, which may have weakened the performance of this score to predict mortality. The strength of this study is the large number of patients, with data relatively uniformly collected at only one site.

## Conclusion

The results support that male sex, age, cognitive impairment, and residential status are important predictors of mortality. The study also found an apparent “obesity paradox” where the mortality was lowest in patients with a BMI of 30–35, but this finding may also be confounded by unmeasured factors such as frailty, sarcopenia, beta-blocker use and other unmeasured comorbidity.

Models including ACCI, NHFS and OFS had a similar ability to predict 1-year mortality, while a model including ASA had a significantly lower predictive ability than ACCI and NHFS.

## Data Availability

Due to data protection issues we are not allowed to share data. Aggregated data may be provided on request.

## References

[1] Katsoulis M, Benetou V, Karapetyan T, Feskanich D, Grodstein F, Pettersson-Kymmer U, et al. Excess mortality after hip fracture in elderly persons from Europe and the USA: the CHANCES project. J Intern Med. 2017;281:300–10. 28093824 10.1111/joim.12586

[2] Downey C, Kelly M, Quinlan JF. Changing trends in the mortality rate at 1-year post hip fracture—a systematic review. World J Orthop. 2019;10:166–75. 30918799 10.5312/wjo.v10.i3.166 PMC6428998

[3] Cheng SY, Levy AR, Lefaivre KA, Guy P, Kuramoto L, Sobolev B. Geographic trends in incidence of hip fractures: a comprehensive literature review. Osteoporos Int a J Establ as result Coop between Eur Found Osteoporos Natl Osteoporos Found USA. 2011;22:2575–86. 10.1007/s00198-011-1596-z 21484361

[4] Kjaervik C, Gjertsen J-E, Engeseter LB, Stensland E, Dybvik E, Soereide O. Waiting time for hip fracture surgery: hospital variation, causes, and effects on postoperative mortality. Bone Jt Open. 2021;2:710–20. 34472378 10.1302/2633-1462.29.BJO-2021-0079.R1 PMC8479844

[5] Kjærvik C, Gjertsen JE, Stensland E, Saltyte-Benth J, Soereide O. Modifiable and non-modifiable risk factors in hip fracture mortality in Norway, 2014 to 2018: a linked multiregistry study. Bone Jt J. 2022;104-B:884–93. 10.1302/0301-620X.104B7.BJJ-2021-1806.R1 PMC9251134 35775181

[6] Bajracharya R, Guralnik JM, Shardell MD, Rathbun AM, Yamashita T, Hochberg MC, et al. Long-term sex differences in all-cause and infection-specific mortality post hip fracture. J Am Geriatr Soc. 2022;70:2107–14. 35415882 10.1111/jgs.17800 PMC9283265

[7] Xu BY, Yan S, Low LL, Vasanwala FF, Low SG. Predictors of poor functional outcomes and mortality in patients with hip fracture: a systematic review. BMC Musculoskelet Disord. 2019;20:1–9. 31775693 10.1186/s12891-019-2950-0 PMC6882152

[8] Charlson M, Szatrowski TP, Peterson J, Gold J. Validation of a combined comorbidity index. J Clin Epidemiol. 1994;47:1245–51. 7722560 10.1016/0895-4356(94)90129-5

[9] Maxwell MJ, Moran CG, Moppett IK. Development and validation of a preoperative scoring system to predict 30 day mortality in patients undergoing hip fracture surgery. Br J Anaesth [Internet]. 2008;101:511–7. 10.1093/bja/aen236. 18723517 10.1093/bja/aen236

[10] Wiles MD, Moran CG, Sahota O, Moppett IK. Nottingham Hip Fracture Score as a predictor of one year mortality in patients undergoing surgical repair of fractured neck of femur. Br J Anaesth [Internet]. 2011;106:501–4. 10.1093/bja/aeq405. 21278153 10.1093/bja/aeq405

[11] Forssten MP, Cao Y, Trivedi DJ, Ekestubbe L, Borg T, Bass GA, et al. Developing and validating a scoring system for measuring frailty in patients with hip fracture: a novel model for predicting short-term postoperative mortality. Trauma Surg Acute Care Open. 2022;7:1–6. 10.1136/tsaco-2022-000962 PMC9472206 36117728

[12] Forssten MP, Cao Y, Mohammad Ismail A, Ioannidis I, Tennakoon L, Spain DA, et al. Validation of the orthopedic frailty score for measuring frailty in hip fracture patients: a cohort study based on the United States National inpatient sample. Eur J Trauma Emerg Surg [Internet]. 2023;49:2155–63. 10.1007/s00068-023-02308-7. 37349513 10.1007/s00068-023-02308-7 PMC10520138

[13] Li J, Li D, Wang X, Zhang L. The impact of body mass index on mortality rates of hip fracture patients: a systematic review and meta-analysis. Osteoporos Int. 2022;33:1859–69. 35551433 10.1007/s00198-022-06415-w

[14] Sheehan KJ, Sobolev B, Chudyk A, Stephens T, Guy P. Patient and system factors of mortality after hip fracture: a scoping review. BMC Musculoskelet Disord [Internet]. 2016. 10.1186/s12891-016-1018-7. 27079195 10.1186/s12891-016-1018-7 PMC4832537

[15] Akram DS, Astrup A V, Atinmo T, Boissin JL, Bray GA, Carroll K, et al. Obesity: Preventing and managing the global epidemic. World Health Organization technical report series. 2000. 11234459

[16] DeLong ER, DeLong DM, Clarke-Pearson DL. Comparing the areas under two or more correlated receiver operating characteristic curves: a nonparametric approach. Biometrics. 1988;44:837–45. 3203132

[17] Goel K, Lopez-Jimenez F, De Schutter A, Coutinho T, Lavie CJ. Obesity paradox in different populations: evidence and controversies. Future Cardiol. 2014;10:81–91. 24344665 10.2217/fca.13.84

[18] Yang TI, Chen YH, Chiang MH, Kuo YJ, Chen YP. Inverse relation of body weight with short-term and long-term mortality following hip fracture surgery: a meta-analysis. J Orthop Surg Res [Internet]. 2022;17:1–12. 10.1186/s13018-022-03131-3. 35473595 10.1186/s13018-022-03131-3 PMC9044716

[19] Stokes A, Preston SH. Smoking and reverse causation create an obesity paradox in cardiovascular disease. Obesity (Silver Spring). 2015;23:2485–90. 26421898 10.1002/oby.21239 PMC4701612

[20] Norwegian Institute of Public Health. Utbredelse av røyking i Norge [Internet]. https://www.fhi.no/nettpub/tobakkinorge/bruk-av-tobakk/utbredelse-av-royking-i-norge/. Accessed 25 Mar 2025

[21] Berg J, Nauman J, Wisløff U. Normative values for body composition in 22,191 healthy Norwegian adults 20–99 years: the HUNT4 study. Prog Cardiovasc Dis. 2024;85:82–92. 38925258 10.1016/j.pcad.2024.06.002

[22] Cederholm T, Bosaeus I, Barazzoni R, Bauer J, Van Gossum A, Klek S, et al. Diagnostic criteria for malnutrition—an ESPEN consensus statement. Clin Nutr [Internet]. 2015;34:335–40. 10.1016/j.clnu.2015.03.001. 25799486 10.1016/j.clnu.2015.03.001

[23] Forssten MP, Mohammad Ismail A, Ioannidis I, Wretenberg P, Borg T, Cao Y, et al. The mortality burden of frailty in hip fracture patients: a nationwide retrospective study of cause-specific mortality. Eur J Trauma Emerg Surg [Internet]. 2023;49:1467–75. 10.1007/s00068-022-02204-6. 36571633 10.1007/s00068-022-02204-6 PMC10229683

[24] Kim HS, Park J-W, Lee Y-K, Yoo J-I, Choi Y-S, Yoon B-H, et al. Prevalence of sarcopenia and mortality rate in older adults with hip fracture. J Am Geriatr Soc. 2022;70:2379–85. 35657018 10.1111/jgs.17905

[25] Li Y, Liu F, Xie H, Zhu Y. Investigation and analysis of frailty and nutrition status in older adult patients with hip fracture. Nutr Clin Pract. 2023;38:1063–72. 37073095 10.1002/ncp.10993

[26] Merchant RA, Seetharaman S, Au L, Wong MWK, Wong BLL, Tan LF, et al. Relationship of fat mass index and fat free mass index with body mass index and association with function, cognition and sarcopenia in pre-frail older adults. Front Endocrinol (Lausanne). 2021;12: 765415. 35002957 10.3389/fendo.2021.765415 PMC8741276

[27] Ismail AM, Ahl R, Forssten MP, Cao Y, Wretenberg P, Borg T, et al. The interaction between pre-admission β-blocker therapy, the Revised Cardiac Risk Index, and mortality in geriatric hip fracture patients. J Trauma Acute Care Surg. 2022;92:49–56. 34252058 10.1097/TA.0000000000003358 PMC8677608

[28] Ahl R, Mohammad Ismail A, Borg T, Sjölin G, Forssten MP, Cao Y, et al. A nationwide observational cohort study of the relationship between beta-blockade and survival after hip fracture surgery. Eur J Trauma Emerg Surg [Internet]. 2022;48:743–51. 10.1007/s00068-020-01588-7. 33507317 10.1007/s00068-020-01588-7 PMC9001555

[29] Gautun H, Syse A. Earlier hospital discharge: a challenge for Norwegian municipalities. Nord J Soc Res. 2017;8:1–17.

[30] Kannegaard PN, van der Mark S, Eiken P, Abrahamsen B. Excess mortality in men compared with women following a hip fracture. National analysis of comedications, comorbidity and survival. Age Ageing. 2010;39:203–9. 20075035 10.1093/ageing/afp221

[31] Hägg S, Jylhävä J. Sex differences in biological aging with a focus on human studies. Elife. 2021;10:1–27. 10.7554/eLife.63425 PMC8118651 33982659

[32] Leer-Salvesen S, Engesæter LB, Dybvik E, Furnes O, Kristensen TB, Gjertsen JE. Does time from fracture to surgery affect mortality and intraoperative medical complications for hip fracture patients? An observational study of 73 557 patients reported to the Norwegian hip fracture register. Bone Jt J. 2019;101-B:1129–37. 10.1302/0301-620X.101B9.BJJ-2019-0295.R1 31474142

[33] Sundet M, Sundin U, Godø A, Sydnes K, Valland H, Sexton J, et al. Use of direct-acting anticoagulants (DOACs) delays surgery and is associated with increased mortality in hip fracture patients. Eur J Trauma Emerg Surg. 2024;50:1851–7. 38713220 10.1007/s00068-024-02532-9 PMC11458687

